# Tolosa-Hunt Syndrome Presenting After COVID-19 Vaccination

**DOI:** 10.7759/cureus.16791

**Published:** 2021-07-31

**Authors:** Tzu Ying Chuang, Karina Burda, Ephrem Teklemariam, Kamran Athar

**Affiliations:** 1 Neurology, Thomas Jefferson University Hospital, Philadelphia, USA; 2 Neurosurgery, Thomas Jefferson University Hospital, Philadelphia, USA

**Keywords:** neuro-ophthalmology, covid-19, neuro-immunology, headache, headache disorders, covid-19 vaccine, autoimmune neurology, neuroinflammation

## Abstract

Coronavirus disease 2019 (COVID-19) infection is associated with a plethora of neurological complications. Newly developed vaccinations targeting the severe acute respiratory syndrome coronavirus 2 (SARS-CoV-2) viral spike protein represent a great epidemiological promise with respect to the resolution of the pandemic. However, vaccinations are not without side effects and, in rare cases, have even been implicated in various autoimmune phenomena. In this report, we describe a case of Tolosa-Hunt syndrome (THS), a granulomatous inflammatory process of the cavernous sinus, occurring in a patient one week after getting COVID-19 vaccination. This rare diagnosis of exclusion must be considered in patients presenting with painful ophthalmoplegia.

## Introduction

Autoimmune phenomena, such as Guillain-Barré syndrome, have been associated with influenza vaccination, while acute disseminated encephalomyelitis has been described in children following vaccination with measles, mumps, and rubella vaccinations [1]. The Vaccine Adverse Event Reporting System (VAERS) lists serious reportable adverse effects after coronavirus disease 2019 (COVID-19) vaccination as events that prompt inpatient hospitalization and persistent incapacity or disruption of the ability to conduct normal life functions. COVID-19 vaccination-associated thrombosis with immune thrombocytopenia has been described with respect to the adenoviral vector-based ChAdOx1 nCov-19 vaccination [2,3]. Myocarditis has been described in young people receiving mRNA-based COVID-19 vaccination [4-6]. To our knowledge, this is the first report of a case of Tolosa-Hunt syndrome (THS) presenting five days after vaccination with an mRNA-based COVID-19 vaccine (mRNA-1273). Despite the reported side effects, the overwhelming benefits of COVID-19 vaccination during this pandemic outweigh the likelihood of potential adverse events given the rarity of post-vaccination sequelae.

## Case presentation

A 45-year-old previously healthy man presented seven days after receiving COVID-19 vaccination with a three-day history of severe left-sided headache, left eye pain with progressive left eye ptosis, decreased vision, and binocular diplopia. His examination was notable for left eye ptosis with an afferent pupillary defect and complete ophthalmoplegia. The remainder of his physical and neurological exam was unremarkable.

Basic lab work was largely unremarkable with the exception of mild hyponatremia (sodium: 125 mmol/L), which later normalized without any intervention. Serum inflammatory workup demonstrated a mildly elevated C-reactive protein of 1.10 mg/dL but was otherwise unremarkable. He had a normal sedimentation rate and thyroid-stimulating hormone levels. Negative tests included anti-nuclear antibody, anti-nuclear cytoplasmic antibody, complement, angiotensin-converting enzyme, anti-smooth muscle, and GQ1B. His A1c was 5.1%. Testing for serum and cerebrospinal fluid (CSF) Lyme, syphilis, and acid-fast bacilli (AFB) was negative. CSF myelin basic protein (MBP) was also negative, and the immunoglobulin G (IgG) index was not elevated. CSF results (WBC 2, RBC 0, protein 44, negative meningitis/encephalitis panel, and negative gram stain and AFB) were not concerning for infection of the central nervous system.

**Figure 1:**
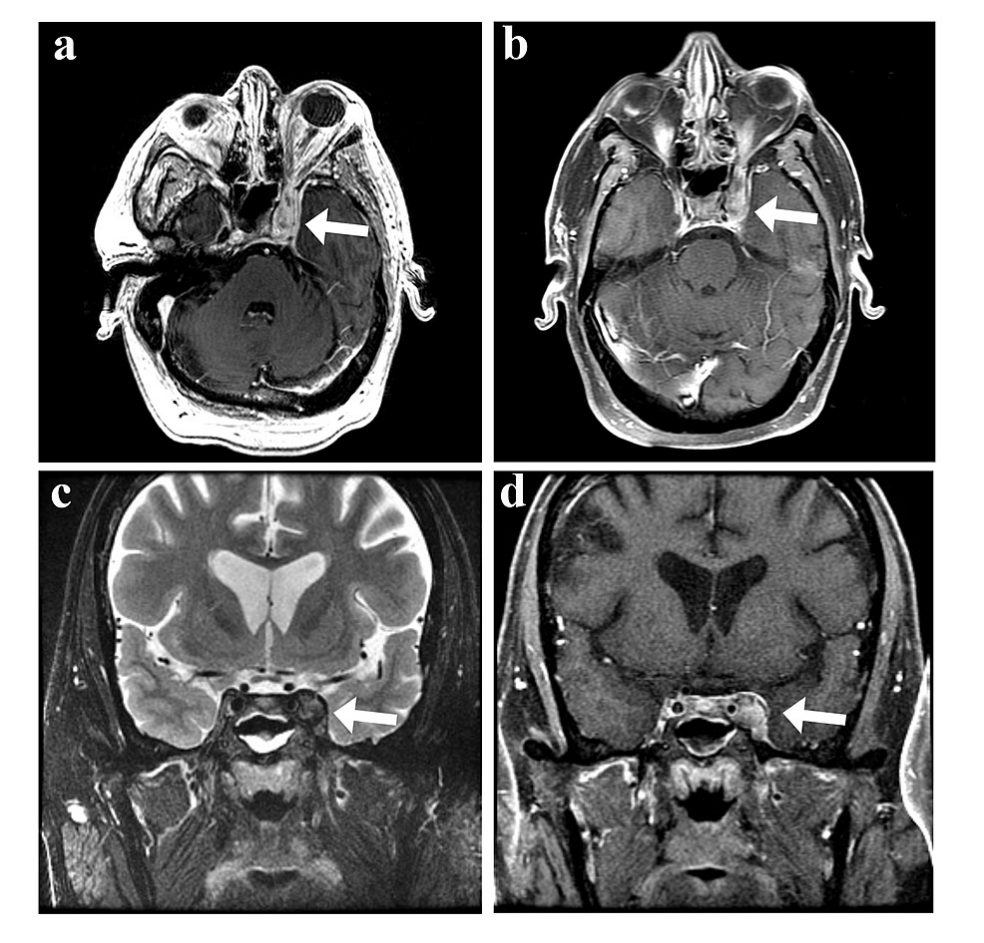
MRI of an inflammatory left cavernous sinus process consistent with Tolosa-Hunt syndrome T2 axial FLAIR (a) and FSE coronal (c) images showing bulky perineural tissue extending into the left cavernous sinus. The perineural tissue has heterogeneous post-contrast enhancement and slightly decreased enhancement centrally consistent with a component of thrombosis on post-contrast T1 axial (b) and coronal (d) images MRI: magnetic resonance imaging; FLAIR: fluid-attenuated inversion recovery; FSE: fast spin-echo

CT of the head demonstrated hyperattenuation of the left cavernous sinus, which was concerning for cavernous sinus thrombosis versus dural-based mass. Contrast-enhanced MRI of the brain and orbits, as well as magnetic resonance angiography (MRA)/magnetic resonance venography (MRV) of the head, demonstrated bilateral perineural enhancement surrounding the optic nerve sheaths (left greater than right). There was also an ill-defined enhancement in the left orbital apex extending into the cavernous sinus suggestive of an inflammatory versus infectious process with a slight decrease in enhancement centrally consistent with possible superimposed thrombosis. The presence of the thrombus was ultimately deemed not definitive (Figure 1). Neurosurgery was consulted for the evaluation of a potential underlying vascular abnormality. On their review of imaging, they were not suspicious of a carotid-cavernous fistula and did not recommend a diagnostic angiogram.

He was initially treated with broad-spectrum antibiotics, which were discontinued after serologic and CSF studies failed to demonstrate evidence of infection. His imaging findings were discussed with neuroradiology and assumed to likely represent an underlying inflammatory process consistent with THS. The patient was treated with one gram of intravenous methylprednisolone for three days. He had significant improvement in pain with minimal improvement in ptosis and eye motility. He was ultimately discharged home on an oral steroid regimen. The patient was seen again in the clinic two months after the initial admission with improvement in his cranial nerve deficits. He continues to follow up with neurology and neuro-ophthalmology for close monitoring of any recurrence.

## Discussion

Painful ophthalmoplegia is associated with a variety of processes localizing to the cavernous sinus, with a frequent focus on cavernous sinus thrombosis as an etiology. However, consideration should also be given to an entity associated with granulomatous inflammation of the cavernous sinus known as Tolosa-Hunt syndrome, a rare diagnosis of exclusion that is remarkably steroid-responsive.

Diagnostic criteria for THS are classified in the International Classification of Headache Disorders, Third Edition (ICHD-3) as episodic pain associated with unilateral headache and paralysis of one or more of the third, fourth, and sixth cranial nerves [7]. It has an estimated incidence of one in a million people. The natural history of THS follows an unpredictable course. Symptoms last from days to weeks and spontaneous remission may occur without the use of systemic corticosteroids [8]. There is a lack of randomized prospective trials on treatment regimens due to the rarity of the condition [9].

The differential diagnoses for the condition include neoplasms (including lymphoma), aneurysm, carotid-cavernous fistula, carotid dissection, primary cavernous sinus thrombosis, infection, vasculitis, and sarcoidosis. CSF test and neuroimaging are essential for excluding other causes of painful ophthalmoplegia. THS remains a diagnosis of exclusion, and clinical symptoms and diagnostic imaging considered together have a high sensitivity and low specificity for THS [10].

## Conclusions

Diagnostic consideration into painful ophthalmoplegia must include a careful history to encompass a wide differential. Given the ongoing pandemic, the history must include both COVID-19 infection and vaccination against it to further elucidate potential post-infectious or autoimmune phenomena. Antecedent COVID-19 vaccination could have been coincidental with idiopathic THS in this patient. THS is listed as an adverse event of special interest (AESI) under VAERS. Due to the rarity of THS, it would be useful to know whether there is an increased incidence of cases associated with either COVID-19 infection or vaccination.

This case further highlights the importance of considering common presentations of an uncommon disease in the evaluation of painful ophthalmoplegia, as this can minimize unnecessary exposure to antibiotics and anticoagulants. Furthermore, early recognition of THS can lead to early symptomatic relief given its steroid-responsive nature.
